# New l-Rhamnose-Binding Lectin from the Bivalve *Glycymeris yessoensis*: Purification, Partial Structural Characterization and Antibacterial Activity

**DOI:** 10.3390/md22010027

**Published:** 2023-12-29

**Authors:** Tatyana O. Mizgina, Irina V. Chikalovets, Tatyana A. Bulanova, Valentina I. Molchanova, Alina P. Filshtein, Rustam H. Ziganshin, Eugene A. Rogozhin, Nadezhda V. Shilova, Oleg V. Chernikov

**Affiliations:** 1 G.B. Elyakov Pacific Institute of Bioorganic Chemistry, Far Eastern Branch of Russian Academy of Sciences, Vladivostok 690022, Russia; ivchik6@mail.ru (I.V.C.); molchanova_val@mail.ru (V.I.M.); alishichka@mail.ru (A.P.F.); 2 Department of Chemistry and Materials, Far Eastern Federal University, Vladivostok 690950, Russia; tanushka.bulanova@mail.ru; 3 Shemyakin-Ovchinnikov Institute of Bioorganic Chemistry, Russian Academy of Sciences, Moscow 117997, Russia; rustam.ziganshin@gmail.com (R.H.Z.); rea21@list.ru (E.A.R.); pumatnv@gmail.com (N.V.S.)

**Keywords:** l-rhamnose-binding lectin, bivalve lectin, hemolymph, microorganism binding, innate immune

## Abstract

In this study, a new l-rhamnose-binding lectin (GYL-R) from the hemolymph of bivalve *Glycymeris yessoensis* was purified using affinity and ion-exchange chromatography and functionally characterized. Lectin antimicrobial activity was examined in different ways. The lectin was inhibited by saccharides possessing the same configuration of hydroxyl groups at C-2 and C-4, such as l-rhamnose, d-galactose, lactose, l-arabinose and raffinose. Using the glycan microarray approach, natural carbohydrate ligands were established for GYL-R as l-Rha and glycans containing the α-Gal residue in the terminal position. The GYL-R molecular mass determined by MALDI-TOF mass spectrometry was 30,415 Da. The hemagglutination activity of the lectin was not affected by metal ions. The lectin was stable up to 75 °C and between pH 4.0 and 12.0. The amino acid sequence of the five GYL-R segments was obtained with nano-ESI MS/MS and contained both YGR and DPC-peptide motifs which are conserved in most of the l-rhamnose-binding lectin carbohydrate recognition domains. Circular dichroism confirmed that GYL is a α/β-protein with a predominance of the random coil. Furthermore, GYL-R was able to bind and suppress the growth of the Gram-negative bacteria *E. coli* by recognizing lipopolysaccharides. Together, these results suggest that GYL-R is a new member of the RBL family which participates in the self-defense mechanism against bacteria and pathogens with a distinct carbohydrate-binding specificity.

## 1. Introduction

Lectins are non-enzymatic and non-immunoglobulin proteins that bind with sugars or glycans and accomplish various functions in a number of cellular processes [1]. They are also involved in innate immunity and possess antimicrobial properties through their binding and interaction with microbial glycoconjugates [2]. Lectins have been grouped into many families based on their carbohydrate recognition domains (CRDs). Animal lectins are characterized by different structures, carbohydrate specificities and physiological roles [3]. Although invertebrate lectins have been intensively studied recently, information on their isolation sources, structure, and properties is limited compared to lectins from higher animals and terrestrial plants. Structural and functional studies of lectins isolated from marine invertebrates will expand the understanding of the diversity of lectins and the basics of their functioning [4].

Invertebrate innate immune recognition is a fundamental and core reaction to sense invaders and trigger the following immune protection. So far, various pattern recognition receptors (PRRs) have been reported to induce immune defense in mollusks by recognizing conserved structures of different pathogens, called pathogen-associated molecular patterns (PAMPs) [5]. Among the known PRR lectins from marine invertebrates have been demonstrated to play key roles in various immune events. These mechanisms may be very effective in bivalves, a group of marine fauna vulnerable to various pollutants and aquatic microorganisms, as they are sedentary filtering organisms. Most likely, bivalves have developed mechanisms to avoid excessive immune reactions and to exert strong control of their inflammatory responses [6]. Invertebrate lectins are characterized by different structures, size, molecular organization, carbohydrate specificity and classified into galectins, C-type lectins, fucolectins, pentraxins and rhamnose-binding lectins (RBLs) [7,8].

RBLs form a family of proteins described in many animals, both vertebrates and invertebrates and include proteins that have diverse biological functions, including fertilization, cell proliferation, cytotoxicity and innate immunity [1,9]. The ability to recognize and suppress the growth of various harmful microorganisms and cancer cells makes RBL potential targets for use in medicine [10,11,12].

The first and best characterized RBL is sea urchin egg lectin (SUEL), specific for d-galactosides, isolated about 30 years ago from the eggs of the Japanese purple sea urchin *Anthocidaris crassispina* [13]. However, further research revealed its stronger affinity for l-rhamnose. This affinity can be attributed to the similar orientation of hydroxyl groups at C2 and C4 in the pyranose rings of l-rhamnose and d-galactose.

More recently, RBLs have been found in abundance in the serum, embryos and eggs of teleost fishes [14,15,16], and other aquatic invertebrate species like ascidians [17,18] and echinoderms [19,20], where they play important role in immunomodulation. RBLs are composed of one or multiple characteristic CRDs particularly recognizing l-rhamnose or d-galactose without Ca^2+^ dependence. The approximately 100 amino acids-long CRD contains characteristic peptide motifs such as YGR, DPC, and KYL, and displays four disulfide bridges through eight highly conserved cysteine residues resulting in the characteristic topology with a unique structural α/β fold [21,22]. Little is known about RBL from marine bivalves, with the exception of a galactose-binding lectin isolated from the oyster *Pteria penguin*, which was poorly inhibited by l-rhamnose but was homologous to some RBLs [23].

In our previous study, the C-type lectins GYL and GYLman were identified from the hemolymph of mollusk *G. yessoensis*. They have a wide spectrum of microbial binding, including Gram-positive (*Bacillus subtilis*, *Staphylococcus aureus*) and Gram-negative bacteria (*Escherichia coli*, *Vibrio proteolyticus*) and possibly participate in the immune defense of the *G. yessoensis* against bacterial attacks [24,25,26]. Considering the large number of bacteria in the aquatic environment, there should be other lectins to protect the mollusk from various pathogens. The main objectives of this study were the isolation, study of biochemical properties, carbohydrate-binding specificity and characterization the potential role in immune response of the new l-rhamnose binding lectin from the hemolymph of the mollusk *G. yessoensis.*

## 2. Results and Discussion

### 2.1. Purification of G. yessoensis Lectin

To obtain hemolymph, we used bivalves *G. yessoensis* collected in the Sea of Japan. Hemolymph samples collected from adductor muscles were centrifuged to obtain pure supernatant (plasma), which was used for lectin isolation and purification.

**Figure 1:**
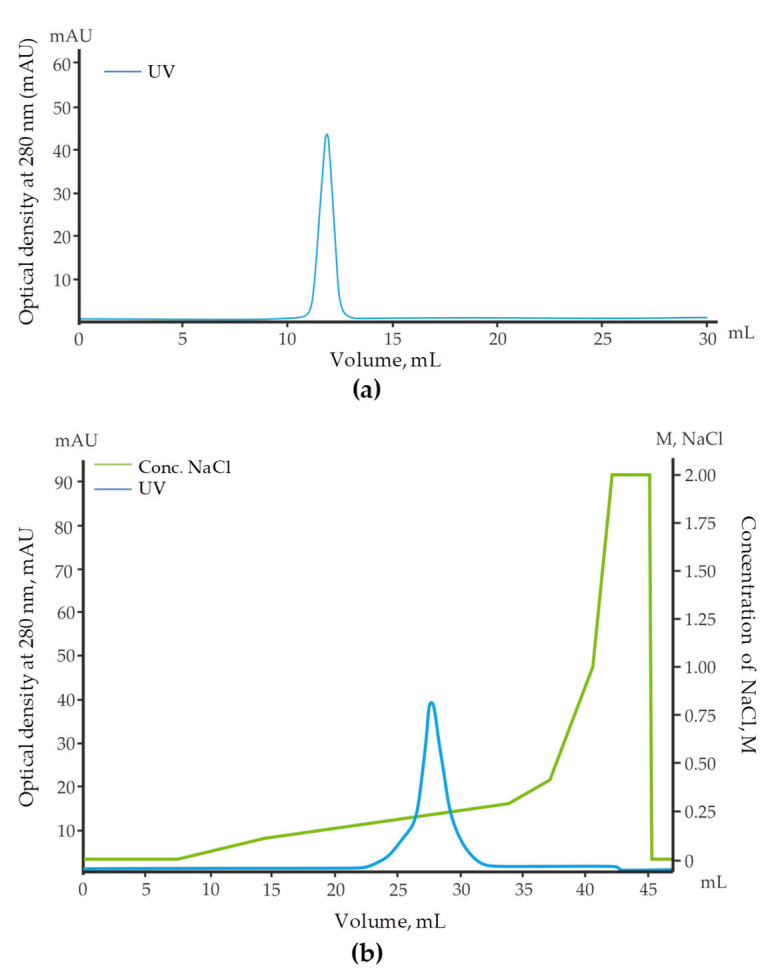
(**a**) Size exclusion chromatography of lectin on Superdex 75. (**b**) Anion-exchange chromatography of lectin on SOURCE 15Q.

Affinity chromatography techniques are the most efficient lectin isolation methods [1,27]. In this study, lactose was selected as a ligand for affinity sorbent synthesis because in preliminary study it was shown the strongest inhibition of the hemolymph hemagglutination by saccharides possessing the same configuration of hydroxyl groups at C-2 and C-4, such as l-rhamnose, raffinose, d-galactose and lactose. As a rule, RBLs are isolated on affinity sorbents such as rhamnosyl-sepharose [28], or galactosyl-agarose [14]. However, the use of lactosyl-sepharose or lactosyl-agarose for this purpose also allowed to isolate several lectins specific not only to lactose, but also to rhamnose [20]. The protein eluted from lactosyl-sepharose CL-4B with 0.2 M l-rhamnose was further purified by gel filtration on a Superdex 75 Increase 10/300 column (Figure 1a) and anion-exchange chromatography on a SOURSE 15 Q column (Figure 1b).

**Figure 2:**
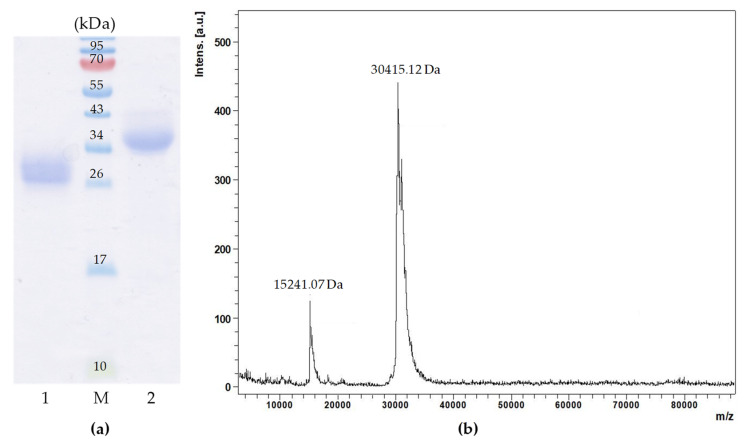
(**a**) SDS-PAGE of GYL-R. Protein bands were stained with Coomassie Brilliant Blue R-250 reagent. Lanes: M—molecular weight markers (kDa); 1—GYL-R in non-reducing conditions (without DTT); 2—GYL-R in reducing conditions (with DTT). (**b**) Molecular mass determination of GYL-R via MALDI-TOF mass spectrometry.

Purified lectin, named GYL-R, showed a single band (approximately 27 kDa) in the absence of a reducing agent DTT, and approximately 32 kDa in reducing conditions (Figure 2a).

Most likely, GYL-R is monomer and, like other representatives of rhamnose-specific lectins, has four internal disulfide bonds in its structure. Under reducing conditions, the monomer undergoes conformational changes due to the breaking of disulfide bridges and acquires a more “loose” structure, which causes a slowdown in migration and leads to an increase in the apparent molecular weight. This behavior under electrophoresis conditions is characteristic of RBLs and has been described for many lectins of this class [28,29]. The GYL-R molecular mass determined by MALDI-TOF mass spectrometry was 30,415.12 Da, that corresponds to the lectin isolated in the individual state, a single-charged ion (M + H)^+^. Peak 15,241.07 Da—double-charged ion (M + H)^++^ (Figure 2b and Figure S1, File S1). This corresponds to the range of molecular weights of known RBL, for example, the molar mass of l-rhamnose-binding lectin SUL-1 from the sea urchin *Toxopneustes pileolus* is 30.5 kDa [30], Chinook salmon roe lectin had a molecular mass of 30 kDa [9] and lectin from a hematophagous insect *Rhodnius prolixus* possesses a mature protein of 34.6 kDa [21].

### 2.2. Hemagglutinating Activity and the Carbohydrate Specificity

The isolated lectin agglutinated human (blood type O) and rabbit erythrocytes. Treatment of erythrocytes with trypsin was shown to enhance the hemagglutination, GYL-R strongly agglutinated enzyme-treated rabbit erythrocytes with the highest hemagglutinating activity (HA) 2048 titer, moderately agglutinated human (blood type O) and weakly agglutinated the remaining human red blood cells (Table 1). Mild enzymatic treatment of erythrocytes can expose hidden glycans on erythrocytes surface and allow access to certain lectins. Trypsin treatment of erythrocytes alters the sialic acid content of erythrocyte sialoglycoproteins. Also, trypsin induces proteolysis of glycophorin A resulting in conformational changes and appearance of new binding sites for lectins. Further formation of carbohydrate-protein bridges between neighboring cells leads to a higher aggregation of erythrocytes [31].

Carbohydrate specificity of the isolated lectin was determined by hemagglutination-inhibition test. A set of mono-, oligo- and polysaccharides, glycoproteins were used as inhibitors of lectin activity.

As can be seen from Table 2, for GYL-R the best inhibitor is l-rhamnose. d-galactose and its derivatives which possess the same hydroxyl group orientation at C-2 and C-4 of the pyranose ring structure of l-rhamnose, also showed inhibitory effects. Therefore, OH groups in this position are important for the interaction of the carbohydrate-binding site of the lectin with these sugars.

Determination of the specificity of GYL-R to natural carbohydrate ligands was carried out using the Glycan microarray method. In total, 378 oligosaccharides were found in N-, O-glycans and glycosphingolipids of mammalian tissues, as well as 229 bacterial polysaccharides were used. Selective GYL-R (50 µg/mL) binding entities, including glycan structure and binding signals in relative fluorescence units (average RFU) in decreasing order are presented in Table 3. GYL-R highly specifically bound the monosaccharide L-Rha and glycans containing the α-Gal residue in the terminal position. In addition to rhamnose binding, some RBLs showed specific binding affinity with trisaccharide Galα1-4Galβ1-4Glc (globotriose, Gb3) which is a tumour-associated glycosphingolipid, highly presented in a plethora of human cancers [32]. These lectins exhibit an antiproliferative effect against Raji Burkitt’s lymphoma cells, on the surface of which Gb3 is expressed. SAL, a lectin isolated from the caviar of *Silurus asotus* fish belonging to RBLs, induces G0/1 phase cell cycle arrest in Raji cells by binding to Gb3 [12], lectin CSL3 from the eggs of the chum salmon *Oncorhynchus keta* used Gb3 as a cellular receptor to evoke apoptosis [33]. Although lectins have high specificity for monosaccharides, their affinity is weaker compared to polyvalent glycotopes, which, having suitable sugar sequences and geometry, significantly increase affinity for lectins.

Among the oligosaccharides, the strongest binding of GYL-R was found when interacting with the branched tetrasaccharide Galα1-4(Fucα1-2)Galβ1-4GlcNAcβ. Selective high affinity binding to this glycan over other α-Gal terminal oligosaccharides was also achieved by the presence of a fucose residue at the non-reducing end. The same high binding affinity is observed in the presence of substituents in neighboring sugars, such as NAc (N-acetyl). For example, binding to Galα1-4Galβ is 6 times weaker than to Galα1-4GalNAcα and GYL-R binds to Galα1-4Galβ1-4Glcβ (Gb3) 20 times weaker than with trisaccharide Galα1-4Galβ1-4GlcNAcβ and 50 times weaker than with l-Rha. Thus, the glycoarray data confirm that GYL-R binds not only l-Rha, but also Galα1-4 terminated glycans, which is characteristic of RBL family of lectins.

### 2.3. Physico Chemical Characterization of Lectin

#### 2.3.1. The Thermal and pH Stability of GYL-R

**Figure 3:**
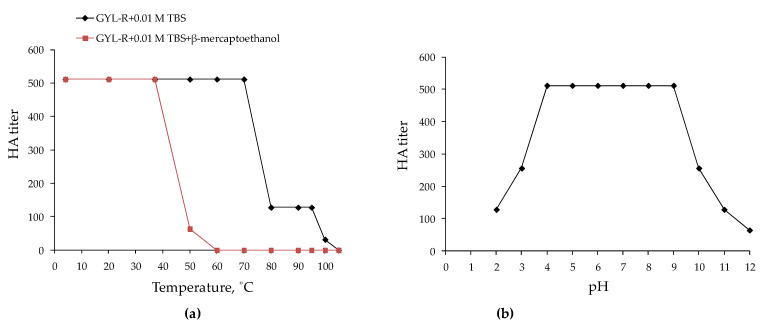
Effects of temperature (**a**) and pH (**b**) on the hemagglutination activity of GYL-R.

The GYL-R solution retained its initial activity up to 70 °C and kept its HA up to 105 °C; however, the activity was completely lost at 110 °C (Figure 3a). Most marine invertebrate lectins are inactivated at lower temperatures. Even two lectins earlier isolated from the same hemolymph of the *G. yessoensis* were maximally active in the range of low temperatures from 4 to 20 °C, which corresponds to the temperature range of the mollusk habitat and is characteristic of most known invertebrate lectins [24,25]. Preservation of hemagglutinating activity at such high temperatures for proteins is characteristic of rhamnose-specific lectins. Thus, PPL, a rhamnose-specific lectin from the oyster *P. penguin*, completely retained its hemagglutinating activity after heat treatment at 60 °C and lost half of its activity after heating at 70 °C. Complete inactivation of PPL required heating at 100 °C for 40 min [23]. Similarly, complete inactivation of the rhamnose-specific lectin DlRBL from the plasma of the sea bass *Dicentrarchus labrax* was achieved by incubating the sample for 30 min at 100 °C [14]. These properties are most likely due to the peculiarities of the amino acid composition of RBLs, namely, the presence of eight conserved cysteines that form four disulfide bonds that stabilize the tertiary structure of the protein and prevent its unfolding upon heating. This assumption was confirmed when the GYL-R solution was heated in the presence of β-mercaptoethanol: the temperature of inactivation of hemagglutination activity decreased significantly and amounted to 60 °C.

After exposing GYL-R to various pH values for 1 h, we noted that the isolated lectin was pH stable (Figure 3b). GYL-R activity was maximum in the pH range from 4.0 to 9.0 and dropped to pH 2.0–3.0 and 10.0–12.0. The minimum activity of the lectin was established at pH 12.0; the pH value of inactivation was not established. It is possible that the stability of hemagglutination activity in such a wide range of pH values is also due to the stabilization of the lectin molecule by disulfide bridges. For bivalves lectins such pH stability of hemagglutination activity is not typical. For example, the galactose-specific lectin from the scallop *Patinopecten yessoensis* is completely inactivated at pH 4.0 and 11.0 [34].

#### 2.3.2. Dependence of the Lectin Activity on the Concentration of Ca^2+^ Ions

Like for most RBLs, the presence or absence of the Ca^2+^ ions was not influenced on lectin activity [7]. No appreciable change was seen in the hemagglutinating activities of GYL-R after treatment with 0.1 M EDTA, whereas no enhanced activity was observed by the addition of Ca^2+^ ion.

### 2.4. Structural Studies by Circular Dichroism (CD)

**Figure 4:**
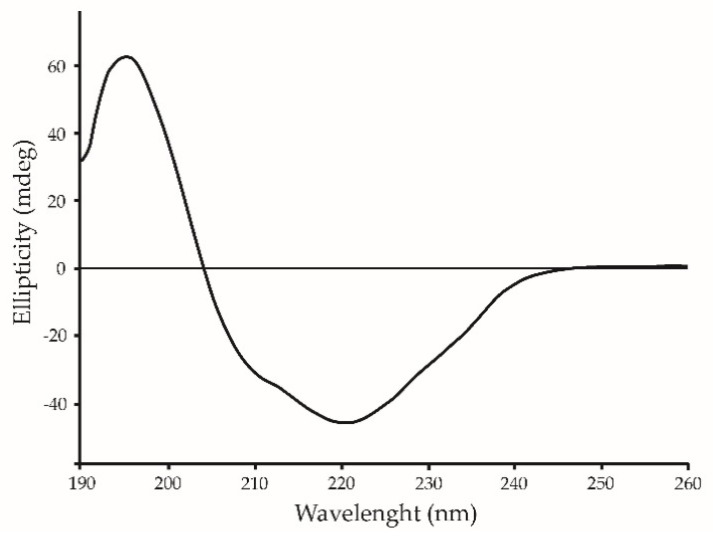
Secondary structure of GYL-R was measured by Far-UV CD spectrum (260 nm–190 nm) with protein concentration of 20 µM at 25 °C.

The content of secondary structure of lectin was studied by CD spectroscopy. The shape of the CD spectra in the far UV region (190–260 nm) and the calculation of the content of protein secondary structure elements using the CDPro software package using the Sreerama method [35] showed that GYL-R belong to α/β-structured proteins (Figure 4). This is a characteristic of rhamnose-specific lectins, since the overall spatial organization of the RBL domain has a β-sandwich structural arrangement with two antiparallel sheets consisting of five β-strands each, a single long α-helix, and one small helical element [21].

### 2.5. Amino Acid Sequencing of GYL-R

To establish the N-terminal sequence, an automated Edman degradation of the N-terminal GYL-R fragment was performed. As a result, a peptide sequence of 15 amino acid residues long was obtained: VNNXQNVDXDSWPFE (X indicates an unidentified amino acid residue).

To establish the complete amino acid sequence the GYL-R was digested with trypsin and analyzed with nano-ESI-MS/MS, which gave multiple peptide sequences (Figure S2). Five major peptides were chosen and after the protein sequences in the protein BlastX (http://www.ncbi.nlm.nih.gov/BLAST/ accessed on 18 July 2023) they showed no significant homology with other lectins listed in the BLAST (Table 4). NCBI Conserved Domain Search program has identified two motifs characteristic of RBLs. Conserved (YGR-motif)-(AN)YGR(TD)- and (DPC-motifs)-DPCXGT(Y)KY(L), which are located in the N- and the C-terminal regions of each domain, respectively, remain almost in all carbohydrate-binding domains of the RBLs [16,30].

The resulting N-terminal GYL-R fragment matches well with the last peptide identified by the nano-ESI-MS/MS method (Table 4). Thus, the resulting peptides can be used to design primers to obtain the complete amino acid sequence of the lectin in the subsequent research.

### 2.6. Antimicrobial Activity

**Figure 5:**
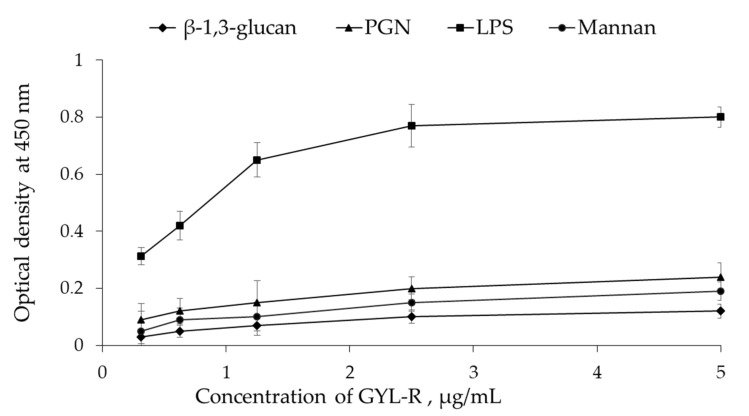
Interaction of GYL-R with PAMP adsorbed on a 96-well plate, determined by the ELLA. Data are presented as mean ± SD (*n* = 3).

The most important feature of lectins is the recognition and agglutination of cells due to non-covalent binding of specific carbohydrate ligands on the cell surface known as pathogen associated molecular patterns (PAMP). The dose-dependent PAMP binding activity of lectin was measured by ELLA using conjugate GYL-R-HRP. GYL-R preferentially bound LPS but had a little binding activity toward PGN, β-1,3-glucan and mannan (Figure 5).

**Figure 6:**
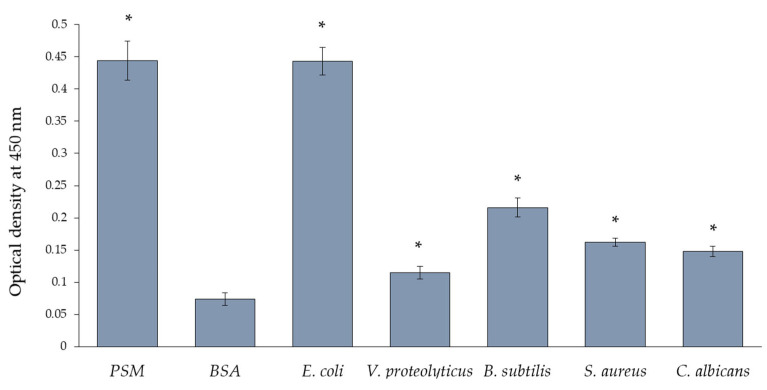
GYL-R binding to microorganisms, determined by ELLA. PSM (porcine stomach mucin)—positive binding control; BSA (bovine serum albumin)—negative binding control. Data are presented as mean ± SD (*n* = 3). Significant difference (*p* < 0.05) with the negative control is indicated with asterisk.

Direct interactions of lectin with bacterial cells were assessed by the same method. It was found that GYL-R highly specifically interacted with the Gram-negative bacterium *E. coli* and had minimal affinity for other microorganisms (Figure 6). This interaction was inhibited by the addition of the specific sugar l-rhamnose and the addition of d-glucose did not affect the binding activity of the lectin with microorganisms.

These data correlate well with the previous experiment. RBL are characterized by interaction with LPS, containing l-rhamnose and d-galactose residues in the O-specific polysaccharide of the cell wall of some *E. coli* strains [36].

The RBL of the bivalve mollusk *P. penguin*—PPL showed antibacterial activity only against the Gram-negative bacterium *E. coli* [23], RBLs from *Oncorhynchus mykiss* trout (STL1, STL2, STL3) bind to LPSs of different serotypes of the Gram-negative bacterium *E. coli*. Furthermore, STL agglutinated a Gram-positive bacterium and bound to its lipoteichoic acid (LTA). The interactions were inhibited by l-rhamnose. These results indicated that STLs can recognize various PAMP [37]. Of all the PAMPs studied GYL-R interacted most effectively with LPS O:111 from *E. coli*, which contains a galactose residue in its structure [38]. The binding activity of a lectin directly depends on its carbohydrate specificity, namely the ability of GYL-R to highly specifically recognize l-rhamnose and d-galactose. The interaction of the GYL-R with cell wall polysaccharides of *E. coli* K12 is probably due to the presence of l-rhamnose in the O-antigen of this bacterium, which is confirmed by the inhibition of binding by this monosaccharide [39]. Lectin binding to *Vibrio proteolyticus* is significantly worse than to *E. coli*, possibly due to the absence of l-rhamnose and d-galactose in the O-antigen LPS of this Gram-negative bacterium.

GYL-R binding to all Gram-positive bacteria is poor since they possess a peptidoglycan layer composed of repeating units of GlcNAc and MurNAc linked by short polypeptide chains [40]. It is well known that the main components of the yeast cell wall are mannoproteins and chitin, so lectin binding to *Candida albicans* is very low [41,42]. Rhamnose is present in all plants, is uncommon in invertebrates and vertebrates but is a common component of the cell wall and capsule of many pathogenic bacteria. Rhamnose recognition may provide an alternative way for pathogen detection and inhibition in the future [43,44].

**Figure 7:**
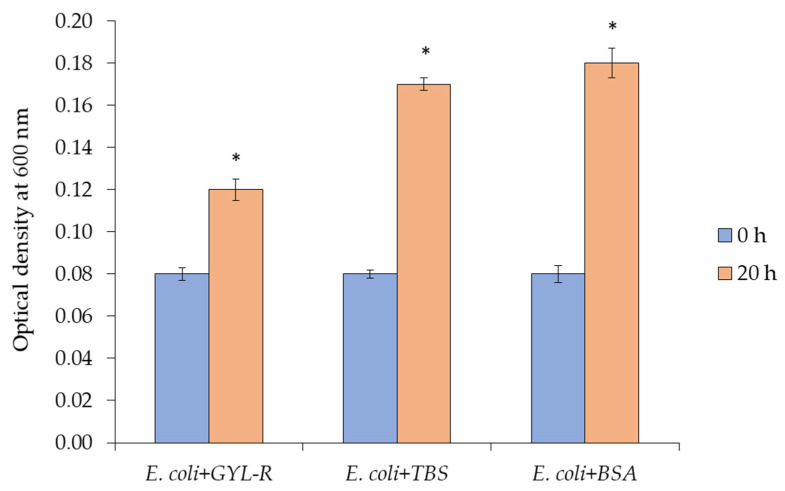
Growth of bacterial cells in absence and presence GYL-R. BSA is negative binding control. Data are presented as mean ± SD (*n* = 3). Significant difference (*p* < 0.05) from the control is indicated with asterisk.

Due to their multidomain structure, many lectins not only bind specific ligands on the cell surface but also agglutinate them, thus inhibiting their further growth [37]. Bacterial cell growth was established in the absence and presence of lectin. GYL-R was able to suppress the growth *E. coli* in a liquid medium but not of other microorganisms tested: *S. aureus*, *B. subtilis*, *V. proteolyticus*, *C. albicans*, corroborating the results described above (Figure 7).

Due to its glycan specificity, GYL-R possessed similar antimicrobial properties as other notable RBLs [37], though the mechanism of action and biological function of the lectin remain unknown. Nevertheless, this lectin must play some role in the self-defense system of *G. yessoensis*, since the lectin is able to highly specifically recognize rhamnose-containing components of the cell walls of pathogens, interacting with Gram-negative bacteria *E. coli* and inhibiting bacterial growth. RBLs play an important role in the innate immune response of marine invertebrates, but the corresponding immune mechanism still requires further study.

## 3. Materials and Methods

### 3.1. Materials

Monosaccharides were obtained from Merck (Darmstadt, Germany). Porcine stomach mucin type III (PSM), bovine serum albumin (BSA), fetuin (Fet), asialofetuin (dsFet), thyroglobulin, and trypsin were purchased from Sigma-Aldrich (St. Louis, MO, USA). LPS from *E. coli* serotype 0111:B4, peptidoglycan (PGN) from *Staphylococcus aureus*, β-1,3-glucan from *Euglena gracilis*, and mannan from *Saccharomyces cerevisiae* were purchased from Sigma-Aldrich (St. Louis, MO, USA). Centricon Ultracel YM-10 was purchased from Millipore (Carrigtwohill, County Cork, Ireland). Human erythrocytes were obtained as outdated red cell concentrates from the Centre of Blood Utilization (Vladivostok, Russia). Rabbit erythrocytes were obtained from the vivarium of the G.B. Elyakov Pacific Institute of Bioorganic Chemistry (Vladivostok, Russia). PageRuler Prestained Protein Ladder standard proteins used for apparent molecular mass estimation by SDS-PAGE were purchased from Thermo Fisher Scientific (Vilnius, Lithuania). Coomassie Brilliant Blue R-250 was purchased from Sigma-Aldrich (St. Louis, MO, USA). PVDF membrane and Trans-Blot Turbo system were purchased from Bio-Rad (Singapore). EZ-Link Sulfo-NHS-Biotinylation Kit was purchased from Thermo FS (Illinois, IL, USA). Gram-positive (*S. aureus* KMM 434, *Bacillus subtilis* ATCC 6633) and Gram-negative (*E. coli* K12, and *Vibrio proteolyticus* CCUG 20302T) bacteria and the yeast *Candida albicans* KMM 455 were obtained from the Collection of Marine Microorganisms (KMM) of the G.B. Elyakov Pacific Institute of Bioorganic Chemistry (Vladivostok, Russia).

### 3.2. Collection of Hemolymph

Clams of the species *G. yessoensis* (G.B. Sowerby III, 1889, taxonomy ID: 2602923, AphiaID 504500), measuring approximately 50 mm in shell length and weighing 23–25 g, were collected from the Marine Experimental Station of the G.B. Elyakov Pacific Institute of Bioorganic Chemistry in Troitsy Bay, located in Posyet Bay, Sea of Japan (GPS coordinates is 42.625523 N 131.130810 E, collected 3 August 2020). The mollusks were identified by a hydrobiologist based on morphological characteristics. The area is home to the only species of mollusks of the genus Glycymeris [45]. Hemolymph samples were drawn from adductor muscles with a sterile syringe, pooled, and centrifuged at 30,000 rpm for 1 h at 4 °C to clarify the hemolymph. Sodium azide and PMSF were added to prevent bacterial growth and inhibit protease activity, respectively. The resulting clear supernatant, referred to as plasma, was tested for hemagglutinating activity and protein concentration. The plasma was then stored in a frozen state at −20 °C for future use.

### 3.3. Isolation and Purification of Lectin from G. yessoensis (GYL-R)

For the isolation of GYL-R by affinity chromatography, an affinity sorbent was synthesized by immobilization lactose on a of the divinylsulfone-activated Sepharose 4B [46]. The clear supernatant (50 mL) was incubated with lactose-Sepharose 4B gel (50 mL) at 4 °C overnight. Unadsorbed substances were removed by washing the gel with 0.01 M TBS (0.01 M Tris-HCl, 0.15 M NaCl, pH 8.5). The adsorbed substance was eluted with 0.2 M l-rhamnose in 0.01 M TBS. The fractions with significant absorption at 280 nm were collected and dialyzed against distilled water, and then lyophilized. The lyophilisate was dissolved in 0.01 M TBS and was subjected to size exclusion chromatography on a Superdex 75 Increase 10/300 GL column (GE Healthcare Bio-Sciences AB, Uppsala, Sweden) equilibrated in 0.01 M TBS, pH 8.5, using an AKTA pure 25 protein purification systems (GE Healthcare Bio-Sciences AB, Uppsala, Sweden). The peak GYL-R was collected, dialyzed against distilled water, and then lyophilized. The lyophilisate was dissolved in 0.01 M TBS just before anion exchange chromatography on a SOURCE 15Q 4.6/100 PE column (GE Healthcare Bio-Sciences AB, Uppsala, Sweden) equilibrated with 0.01 M Tris-HCl buffer (pH 8.5) and eluted with a linear gradient of 0 to 2.0 M NaCl in the same buffer.

### 3.4. Molecular Mass Determination

The relative molecular mass of the purified GYL-R under denaturing condition was estimated by SDS–PAGE in the presence and absence of 0.01 M dithiothreitol (DTT), followed by staining with Coomassie Brilliant Blue, as described by Laemmli [47].

The molecular mass of GYL-R was further investigated by mass spectrometry with an Ultraflex III MALDI-TOF instrument (Bruker, Germany) equipped with a pulsed UV laser (pulse energy 100 mJ, 337 nm). Sinapinic acid was used as the matrix, mixed with the protein sample in a 1:1 ratio. The analysis was performed in linear mode using cytochrome C (12,361.55 Da) and myoglobin (16,952.55 Da) as external standards.

### 3.5. Preparation of 2% Suspension of Native or Enzyme-Treated Erythrocytes

Erythrocytes were washed five times with 50 volumes of 0.15 M NaCl. After washing, a 2% erythrocyte suspension (*v/v*) was prepared in 0.15 M NaCl and used as native erythrocytes. Trypsin-treated erythrocytes were prepared as follows: one-tenth volume of 0.5% (*w/v*) trypsin solution was added to a 2% native erythrocyte suspension, and the mixture was incubated at 37 °C for 2 h. After incubation, the erythrocytes were washed five times with saline, and a 2% suspension (*v/v*) of trypsin-treated erythrocytes was prepared in saline.

### 3.6. Hemagglutinating Activity (HA) and Inhibition Assay

Hemagglutination assays and hemagglutination inhibition assays were performed as described previously [24]. Briefly, to assay the HA, a sample of GYL-R was two-fold serially diluted with 0.01 M TBS in the microtiter U-plates. To the sample (25 μL) in each well, an equal volume of 2% suspension of native or trypsin-treated erythrocytes was added, and the mixture was agitated. The HA was visually evaluated after 30 min. Titer values were defined as the reciprocal of the highest dilution of lectin that gave visible hemagglutination.

For the hemagglutination inhibition assay, the aqueous solutions of various substances (glycoprotein: 1 mg/mL or sugar: 100 mM) were two-fold serially diluted with 0.01 M TBS. To each sample (25 μL), GYL-R (25 μL, 4 doses of agglutination, 0.01 mg/mL) and 2% erythrocyte suspension (25 μL) were added successively, and the mixture obtained was stirred and kept for 1 h. The minimal concentration of each substance required for complete inhibition was determined.

### 3.7. Determination of Fine Carbohydrate Specificity

The fine carbohydrate specificity of the lectin towards mammalian glycans and bacterial polysaccharides was determined using a glycan microarray constructed by Semiotik LLC (Moscow, Russia). Pure GYL-R was labeled with biotin according to the product manual. The array was composed of more than 600 tested glycans, each in 6 replicates. The amount of biotinylated GYL-R bound to each glycan was determined after adding FITC-labeled streptavidin. Slides were scanned with an InnoScan1100 AL (Innopsys, Carbonne, France) with a 488 nm laser at 100% PMT (photomultiplier) gain and high laser power mode. The obtained data were processed using the software Mapix 7.3.1 and Mapix 8.2.2 (Innopsys, France) and the fixed 100 μm-diameter ring method. Data are reported as the relative fluorescence units (RFU) median of six spot replicates. Signals with fluorescence intensity exceeding the background value by a factor of five were considered significant.

### 3.8. Protein Content Measurements

The concentration of soluble proteins was determined by Bradford assay [48] using bovine serum albumin (BSA) as a standard.

### 3.9. Physical and Chemical Treatments

The effects of pH, temperature, and divalent cations on GYL-R were evaluated, as described previously [25]. Briefly, to study the effect of temperature, aliquots of the lectin (0.05 mL, 1 mg/mL) were incubated at 4, 20, 37, 40, 45, 50, 60, 70, 80, 90, 99 and 110 °C for 30 min, and the hemagglutination was performed after cooling to the room temperature. The GYL-R pH dependence was determined by pre-incubating the aliquots of the lectin samples (0.05 mL, 1 mg/mL) with different pH buffers at 25 °C overnight: 0.1 M glycine buffer (pH 2.0; 3.0), 0.1 M acetate buffer (pH 4.0; 5.0), 0.01 M TBS (pH 8.5; 10.0–12.0). The samples were subsequently dialyzed against 0.01 M TBS pH 8.5 to eliminate the pH effect for 12 h, after which the HA was tested.

To evaluate the effect of metal ions, an aliquot of GYL-R (0.3 mL, 1 mg/mL) was dialyzed against a 20-fold volume of 0.01 M TBS with 0.02 M EDTA 12 h and against 0.01 M TBS overnight, after which the HA was determined. Then, a series of two-fold dilutions of the lectin solution of 0.025 mL in 0.01 M TBS was prepared in the microtiter U-plates with the addition of 0, 0.001, 0.005, 0.01, 0.02 M CaCl_2_. The plate was incubated for 30 min at room temperature and the hemagglutination titer was evaluated again.

### 3.10. Circular Dichroism

Far-UV CD spectrum of GYL-R (20 µM in 0.01 M Tris-HCl, pH 8.5) was recorded using an Jasco J-500A spectropolarimeter (Jasco, Tokyo, Japan) with a quartz cuvette of 0.1 cm path length. CD spectrum was collected in Far-UV range from 260 nm to 190 nm at 25 °C. Each spectrum was an average of 3 scans. The results were expressed as mean residual ellipticity (MRE, [h]) in deg∙cm^2^∙dmol^−1^ which was defined as: millidegrees/(path length in mm times the concentration of protein times the number of residues). The secondary structure content of the spectra was determined by the method of Sreerama using the CDPro software package program [35].

### 3.11. Amino Acid Sequencing of Lectin

#### 3.11.1. N-Terminal Sequence of GYL-R

For the analysis of polypeptides by N-terminal sequencing according to Edman, the GYL-R sample after SDS-PAGE was transferred by electroblotting to a polyvinylidene fluoride membrane (Novex, Tucson, AZ, USA). No thiol protection was performed. The detected GYL-R band was excised and subjected to Edman degradation sequencing using a PPSQ-33A protein sequencer (Shimadzu, Japan) according to the manufacturer’s protocol.

#### 3.11.2. Liquid Chromatography and Mass Spectrometry Data Analysis

Sample preparation. Reduction, alkylation and digestion of the GYL-R were performed as described previously [49] with minor modifications. Briefly, 20 μL of sodium deoxycholate (SDC) reduction and alkylation buffer pH 8.5 contained 100 mM TRIS, 1% (*w*/*v*) SDC, 10 mM TCEP and 20 mM 2-chloroacetamide were added to a 20 µg protein sample. The sample was sonicated in an ultrasonic water bath for 1 min, heated at 85 °C for 10 min, cooled to a room temperature and the equal volume of trypsin solution in 100 mM TRIS pH 8.5 was added in a 1:50 (*w*/*w*) ratio. After overnight digestion at 37 °C, peptides were acidified by 40 µL of 2% trifluoroacetic acid (TFA) mixed with 80 µL of ethyl acetate and loaded on SDB-RPS StageTips contained two 14-gauge SDB-RPS plugs, and the StageTip was centrifuged at 300 g until all solution go through the StageTip (typically 5 min). After washing the StageTips with a 100 µL of 1% TFA/ethyl acetate 1:1 mixture (2 times) and 50 µL of 0.2% TFA, peptides were eluted in a clean tube by 60 µL 50% acetonitrile/5% ammonia mixture using centrifugation at 300× *g*. The collected material was vacuum-dried and stored at −80 °C. Before analyses peptides were dissolved in 2% acetonitrile/0.1% TFA buffer at a concentration of 0.5 µg/µL and sonicated for 1 min.

GYL-R samples were loaded onto a homemade 100 µm × 20 mm trap column, packed with Inertsil ODS3 3 µm sorbent (GLSciences) in the loading mobile phase (2% acetonitrile (ACN), 98% H_2_O, 0.1% trifluoroacetic acid) at 10 µL/min flow and separated at room temperature in a home-packed [50] 100 µm × 300 mm fused-silica column packed with ReproSil-Pur C18AQ 1.9 (Dr. Maisch) into an emitter prepared with a P2000 Laser Puller (Sutter, Novato, CA, USA). Reverse-phase chromatography was performed using an Ultimate 3000 Nano LC System (Thermo Fisher Scientific, Waltham, MA, USA), which was coupled to the Orbitrap Q Exactive Plus mass spectrometer (Thermo Fisher Scientific, Waltham, MA, USA) via a nanoelectrospray source (Thermo Fisher Scientific, Waltham, MA, USA). Water containing 0.1% (*v*/*v*) FA was used as mobile phase A, and ACN containing 0.1% FA (*v*/*v*) and 20% (*v*/*v*) H_2_O as mobile phase B. GYL-R peptides were eluted from the trap column with a linear gradient: 3–6% B for 3 min; 6–25% B for 30 min, 25–40% B for 25 min, 40–60% B for 4 min, 60% B for 3 min, 60–99% B for 0.1 min, 99% B for 10 min, and 99–2% B for 0.1 min, at a flow rate of 500 nL/min. After each gradient, the column was re-equilibrated with A for 10 min. MS data were collected in data-dependent acquisition (DDA) mode. MS1 parameters were as follows: 70K resolution, 350–2000 scan range, max injection time 30 s, AGC target 3 × 10^6^. Ions were isolated with a 1.4 *m*/*z* window, preferred peptide match, and isotope exclusion. Dynamic exclusion was set to 30 s. MS2 fragmentation was carried out in high-energy collisional dissociation (HCD) mode at 17.5K resolution with normalized collision energy (NCE) 29, max injection time 50 s, and AGC target 2 × 10^5^, loop count −10. Other settings: charge exclusion—unassigned, 1, >7.

#### 3.11.3. Data Analysis

MS raw files were analyzed using PEAKS Studio 10.6 build 20201221 (Bioinformatics Solutions Inc., Waterloo, ON, Canada) [51]. Identification of proteins was carried out by searching against the *Mollusca* UniProt FASTA database version 26.02.2021 (438,844 entries) with a carbamidomethyl Cys as a fixed modification and deamidation Asn/Gln and Met oxidation as variable modifications. The false discovery rate for peptide spectrum matches was set to 0.01, as determined by searching a reverse database. Enzyme specificity was set as C-terminal to arginine and lysine, and a maximum of two missed cleavages were allowed in the database search. Peptide identification was performed with an allowed initial precursor mass deviation up to 10 p.p.m. and an allowed fragment mass deviation of 0.02 Da.

The obtained peptide sequences were analyzed using BlastX (http://www.ncbi.nlm.nih.gov/BLAST/, accessed on 18 July 2023).

### 3.12. Biological Activity of GYL-R

In experiments to establish the biological activity of lectin, the following microorganisms were used: Gram-positive bacteria *S. aureus* KMM 434 and *B. subtilis* ATCC 6633, Gram-negative bacteria *V. proteolyticus* CCUG 20302T and *E. coli* K12 and yeast *C. albicans* KMM 455. Cells of all microorganisms were prepared for experiments as follows: the cells were centrifuged at 2000 rpm and washed twice with sterile 0.01 M TBS at pH 8.5. For further experiments, cell suspensions were diluted to an OD_600_ of 0.8–1.0, which corresponds to a number of cells ranging from 2 × 10^8^ to 2 × 10^10^ in 1 mL.

#### 3.12.1. PAMP-Binding Assay

The dose-dependent PAMP-binding activity of GYL-R was measured via the enzyme-linked lectin assay (ELLA). GYL-R-HRP was prepared using the periodate method [52]. The following substances were immobilized on 96-well plates (Nunc, Roskilde, Denmark): *E. coli* O111:B4 LPS, *S. aureus* peptidoglycan, *S. cerevisiae* mannan, and *E. gracilis* β-1,3-glucan. The substances were used at a concentration of 50 μg/mL in 0.01 M TBS at pH 8.5 and were added in triplicate at 100 μL per well. The plates were incubated at 4 °C overnight and at 60 °C for 2 h, and then washed three times with 0.01 M TBS at pH 8.5 containing 0.05% Tween-20 (TBS-T). The wells were blocked with 300 µL of 1% BSA for 2 h at room temperature. A conjugate, GYL-R-HRP, was prepared at a concentration of 0.005 mg/mL (dilution of the original conjugate 1:100). Triplicate of 100 μL of the conjugate were applied to the plate in TBS-T using the method of double dilutions. The plate was incubated for 1 h at 37 °C while stirring and then washed three times with TBS-T. After washing, the bound GYL-R-HRP was detected using TMB (tetramethylbenzidine) as a substrate. TMB substrate was added at 100 μL per well, and the plate was incubated in the dark for 5 min. The reaction was terminated by adding 50 μL of 5% H_2_SO_4_. The optical density at 450 nm was measured using a Synergy H1 microplate reader. (BioTek Instruments, Winooski, VT, USA). Experimental reactions for each sample were run in triplicate.

#### 3.12.2. Interaction of GYL-R with Microorganisms

Suspensions of bacteria and yeast were adsorbed onto a 96-well plate (Nunc, Denmark) in triplicates, with 100 μL per well. The plate was incubated overnight at 4 °C. Afterward, the microorganisms were fixed by heating the plate at 80 °C for 45 min, followed by washing three times with TBS-T buffer. BSA was used as a negative control, PSM as positive control. To block free binding sites, 300 μL of a 1% BSA solution was added to each well and incubated with stirring for 1 h at 37 °C. The plate was then washed as described above. The GYL-R-HRP conjugate was prepared at a concentration of 0.005 mg/mL (1:100 dilution of the original conjugate) and applied to the plate in triplicate of 100 μL in TBS-T using the method of double dilutions. The plate was incubated for 1 h at 37 °C while stirring and washed three times with TBS-T buffer. Enzymatic activity was detected, and the reaction was terminated after color development. The optical density was measured as described above.

#### 3.12.3. Inhibition of GYL-R Binding to Microorganisms

Microorganisms were adsorbed onto a 96-well plate (Nunc, Denmark) as described above. Inhibitors (l-rhamnose (specific), d-glucose (nonspecific)) were prepared at a concentration of 0.2 M in 0.01 M TBS pH 8.5. Using the method of double dilutions, 50 μL of inhibitors were added to each well. Simultaneously, 50 μL of GYL-R-HRP conjugate was added to all wells at an initial concentration of 0.01 mg/mL (1:50 dilution of the original conjugate). The plate was incubated for 1 h at 37 °C while stirring and washed three times with TBS-T buffer. Enzymatic activity was detected, and the reaction was terminated after color development. The optical density was measured as described above.

#### 3.12.4. Growth of Bacterial Cells

Bacterial cell growth was studied according to a published protocol [53]. Bacterial cells prepared as described in Section 3.12. were additionally diluted 20 times. On a 96-well plate, 0.05 mL of cell suspensions and 0.05 mL a lectin-containing solution were added in triplicates to achieve a final concentration of 0.2 mg/mL. Cells in sterile 0.01 M TBS at pH 8.5 were used as controls. The cells were incubated at room temperature while stirring, and the optical density was measured at 600 nm every hour.

### 3.13. Statistical Analysis

Experimental data are presented as mean ± SD. A *p*-value less than 0.05 was considered statistically significant.

## 4. Conclusions

In conclusion, a rhamnose-specific lectin, GYL-R, has been identified for the first time from *G. yessoensis,* and its characteristics have been described. Previously, only one RBL was isolated from bivalves. Interestingly, this mollusk involves distinct lectins in the recognition and binding of different pathogens. Recently, two lectins GYLman and GYL have been identified from *G. yessoensis* in our laboratory, and the roles of these lectins and their synergies are still under investigation. In the present study, GYL-R was shown to bind and inhibit the growth only *E. coli*, which was different from the previously reported lectins. So, these results suggest that *G. yessoensis* may present a specific immune response depending upon the type of immunostimulant present.

Our findings offer novel perspectives on the role of RBL as one of the regulators in non-specific immunity within invertebrates. Knowledge about the innate immune systems of marine invertebrates is currently scarce. Thus, exploring the involvement of lectins in the innate immunity of these organisms is highly promising for gaining valuable insights into the innate immune systems of marine animals.

## Figures and Tables

**Table 1:** Agglutination of erythrocytes by GYL-R.

Type of Erythrocytes	Titer of Agglutination ^a^	
	Native	Trypsinized
Human O	128	512
Human A	Na ^b^	2
Human B	Na	2
Human AB	Na	2
Rabbit	128	2048

^a^ The reciprocal of the of highest dilution of lectin that gave visible hemagglutination; ^b^ Na—No agglutination.

**Table 2:** Hemagglutination-inhibition profile of GYL-R ^a^.

Saccharides	MIC ^b^ mg/mL	MIC ^b^ mM	Relative Potency ^c^
l-Rhamnose	0.002	0.12	1.0
d-Galactose	0.156	0.87	78.0
Rafinose	0.625	1.24	312.5
Lactose	0.313	0.91	156.2
PSM ^d^	0.016	-	8.0
dsPSM ^e^	0.063	-	31.5

^a^ Data shown were checked by three experiments; ^b^ The minimum concentration of inhibitors required for complete inhibition; ^c^ Relative potency of inhibitors was compared with respect to l-Rha (Taken as 1.0); ^d^ Porcine stomach mucin; ^e^ Desialylated porcine stomach mucin. The following substances caused no inhibition when used at 10 mg/mL: d-Glucose, N-acetyl-glucosamine, N-acetyl-galactosamine, d-Mannose, N-acetyl-mannosamine, N-acetyl-neuraminic acid, N-acetyl-glycoloylneuraminic acid, melibiose, ovomucoid, alpha-1-acid glycoprotein, mannan from *Saccharomyces cerevisiae,* Fet, dsFet.

**Table 3:** GYL-R binding to glycans is determined by the glycoarray method.

#	Glycan Structure	Average RFU
20	l-Rhaα	64,931
365	Galα1-4(Fucα1-2)Galβ1-4GlcNAcβ	47,673
266	Galα1-4Galβ1-4GlcNAcβ	24,853
81	Galα1-4GlcNAcβ	17,157
815	Galα1-4GalNAcα	12,517
821	Galα1-4Galβ	2404
387	Galβ1-4GlcNAcβ1-6Galβ1-4GlcNAcβ	1829
223	Galα1-4Galβ1-4Glcβ	1245
78	Galα1-3GalNAcα	1061
97	Galβ1-4GlcNAcβ	735

**Table 4:** Peptide sequences.

Peptide	*m/z* ^a^	z	Mass ^b^
LEYASYGR	479.7357	2	957.4556
YLSVVYTCK	566.7903	2	1131.5635
LEAVNSVFGDPCVGTYK	928.4499	2	1854.8821
EDTLVCELNTDLLSCEER	732.6665	3	2194.9722
VNNCQNVDCDSWPFEYK	725.6359	3	2173.8833

^a^ Experimental data; ^b^ Theoretical value.

## Data Availability

The datasets used or analyzed during the current study are available from the corresponding author on reasonable request.

## Supplementary Materials

The following supporting information can be downloaded at: https://www.mdpi.com/article/10.3390/md22010027/s1, Figures S1 and S2: raw mass spectrum data of GYL-R.
