# Supplementary material for: New l-Rhamnose-Binding Lectin from the Bivalve Glycymeris yessoensis: Purification, Partial Structural Characterization and Antibacterial Activity
Source: Mar Drugs. 2023 Dec 29;22(1):27. doi: 10.3390/md22010027 (PMC10817417; doi:10.3390/md22010027)
Supplement: Supplementary file 1 [file marinedrugs-22-00027-s001.zip › GYL-R Supplementary Materials 2023-12-21.pdf]

marine drugs

Supporting Information

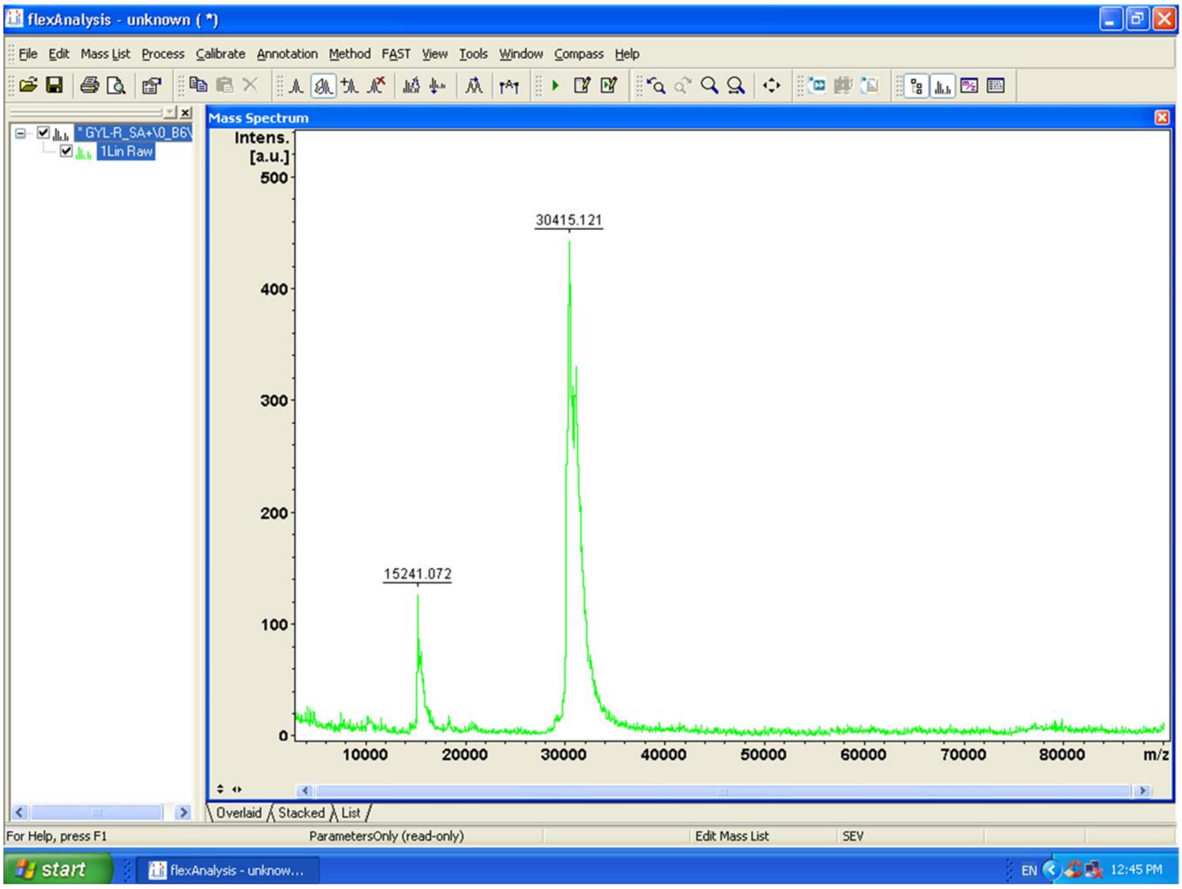

Figure S1. Molecular mass determination of GYL-R via MALDI-TOF mass spectrometry.

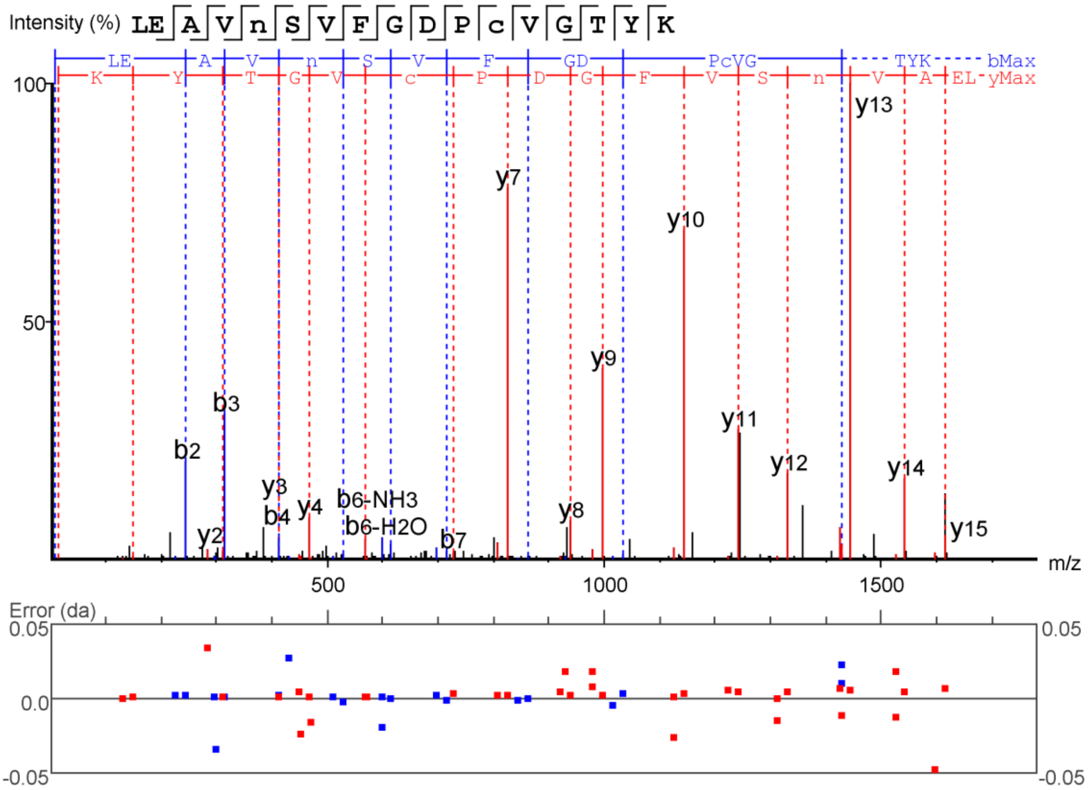
Intensity (%)
LE A V n S V F G D P c V G T Y K
bMax
yMax
y13
y7
y10
y9
b3
y11
b2
y12
y14
y3
b4
y4
b6-NH3
b6-H2O
y8
y2
b7
y15
m/z
500
1000
1500
Error (da)
0.05
0.0
-0.05

(A)

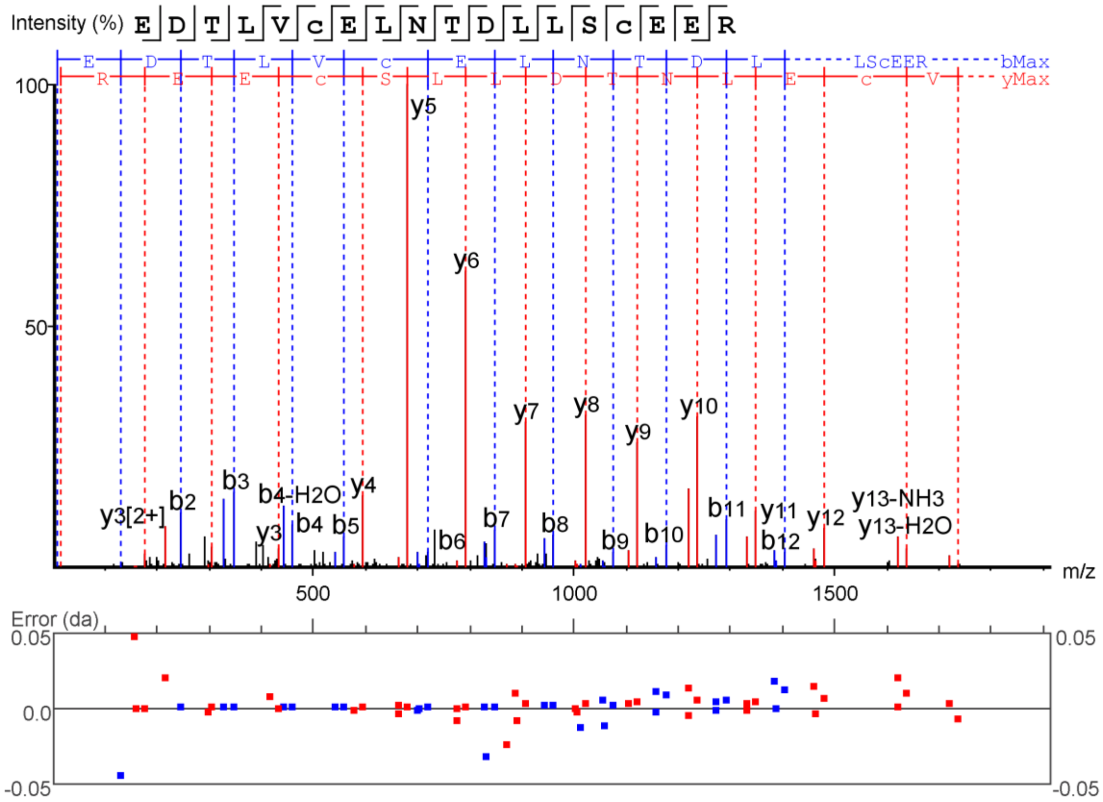
Intensity (%)
E D T L V c E L N T D L L S c E E R
bMax
yMax
y5
y6
y7
y8
y9
y10
b3
y4
b4-H2O
y3[2+]
b2
y3
b4
b5
b6
b7
b8
b9
b10
b11
y11
b12
y12
y13-NH3
y13-H2O
m/z
500
1000
1500
Error (da)
0.05
0.0
-0.05

(B)

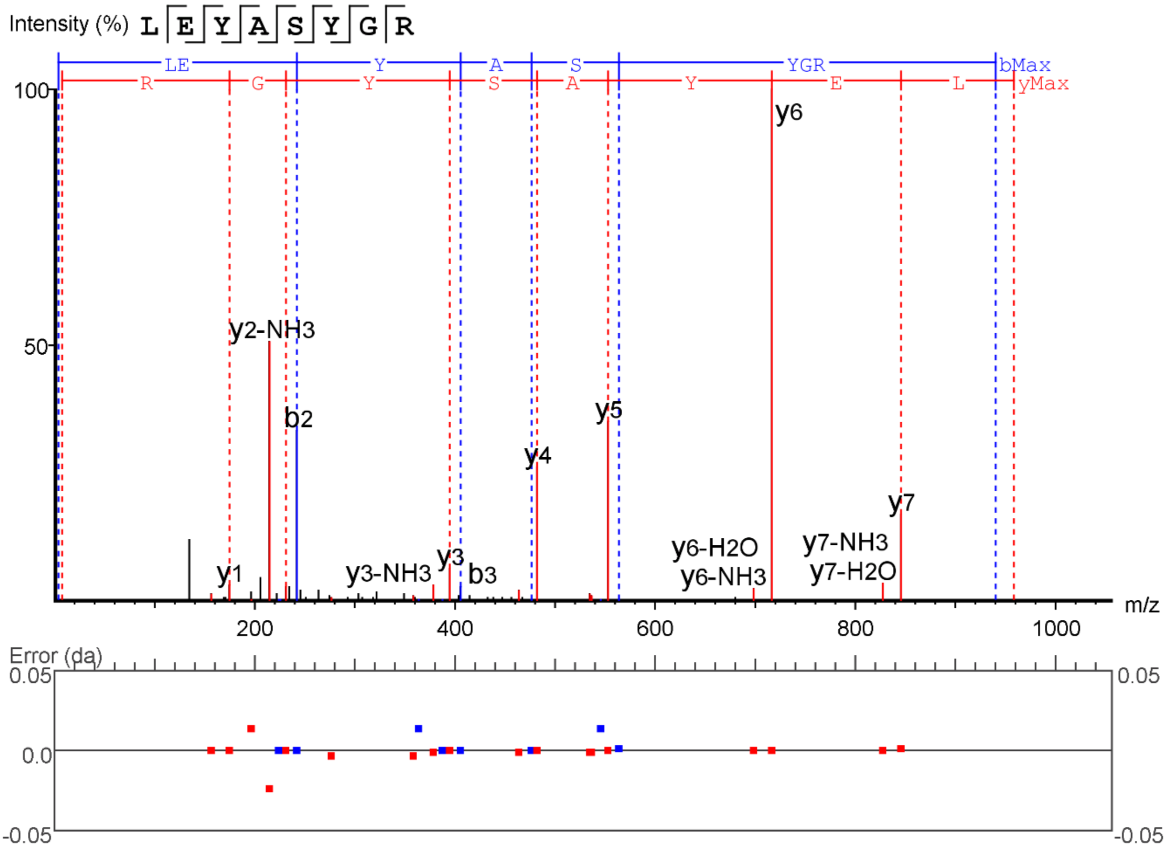
Intensity (%) L E Y A S Y G R
LE
Y
A
S
YGR
bMax
R
G
Y
S
A
Y
E
L
yMax
100
50
y6
y2-NH3
b2
y5
y4
y7
y1
y3-NH3
y3
b3
y6-H2O
y6-NH3
y7-NH3
y7-H2O
m/z
200
400
600
800
1000
Error (da)
0.05
0.0
-0.05

(C)

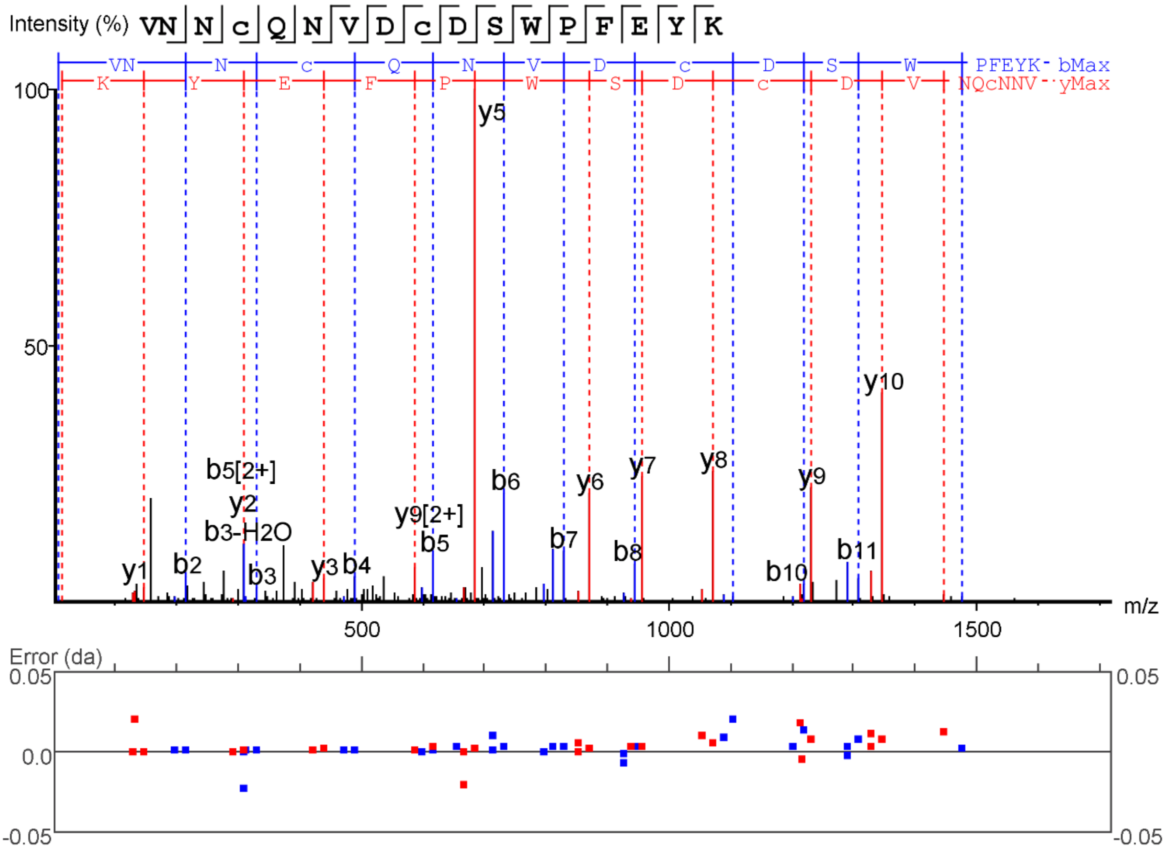
Intensity (%) VN N c Q N V D c D S W P F E Y K
VN
N
c
Q
N
V
D
c
D
S
W
PFEYK
bMax
K
Y
E
F
P
W
S
D
c
D
V
NQcNNV
yMax
100
50
y5
y10
b5[2+]
y2
b3-H2O
b6
y6
y7
y8
y9
y9[2+]
b5
b7
b8
b10
b11
y1
b2
b3
y3
b4
m/z
500
1000
1500
Error (da)
0.05
0.0
-0.05

(D)

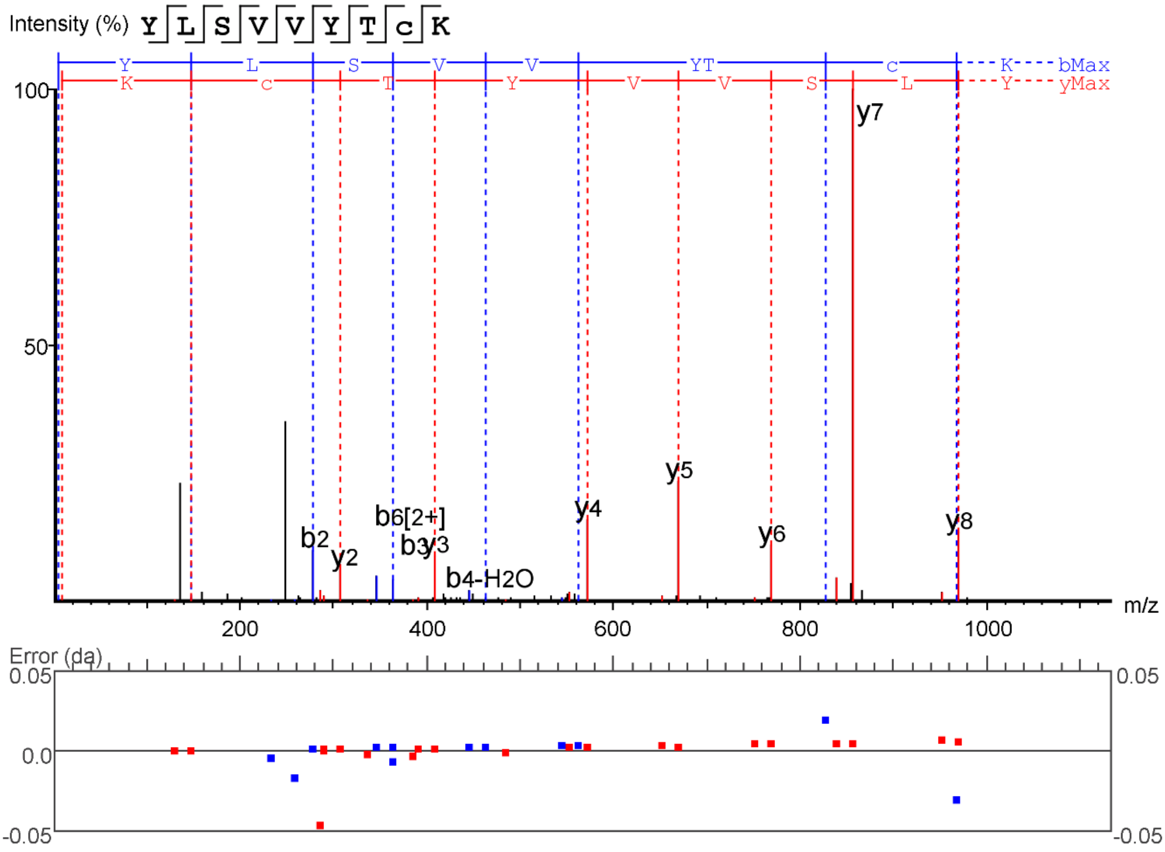

(E)

Figure S2. Spectral data showing fragmentation analysis of peptides of GYL-R. Small letters indicate modified amino acid residues (c – carbamidomethyl Cys, n – deamidation Asn). (**A**) peptide – LEAVNSVFGDPCVGTYK; (**B**) peptide - EDTLVCELNTDLLSCEER; (**C**) peptide – LEYASYGR; (**D**) peptide – VNNCQNVDCDSWPFEYK; (**E**) peptide - YLSVVYTCK.
