# Supplementary figures and images for: New l-Rhamnose-Binding Lectin from the Bivalve Glycymeris yessoensis: Purification, Partial Structural Characterization and Antibacterial Activity
Source: Mar Drugs. 2023 Dec 29;22(1):27. doi: 10.3390/md22010027 (PMC10817417; doi:10.3390/md22010027)

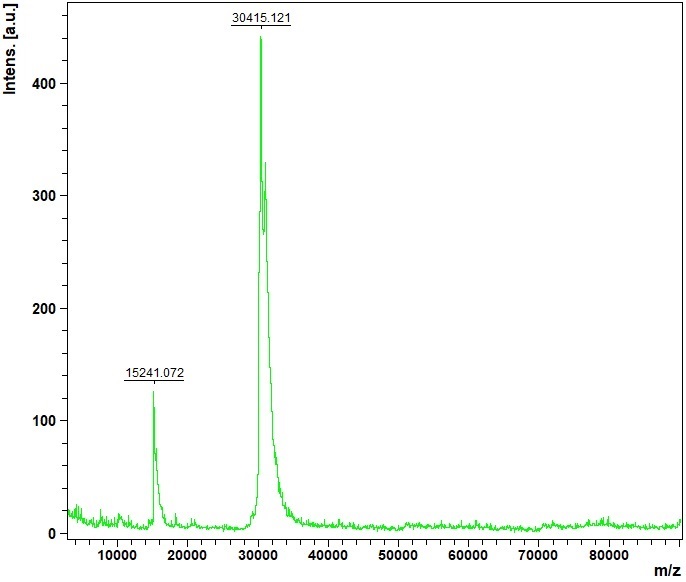

Supplement: Supplementary file 1 [file marinedrugs-22-00027-s001.zip › marinedrugs-2694214.jpg]
